# Soil Fungal Community in Grazed Inner Mongolian Grassland Adjacent to Coal-Mining Activity

**DOI:** 10.3389/fmicb.2021.718727

**Published:** 2021-09-17

**Authors:** Linlin Xie, Yinli Bi, Xianglei Li, Kun Wang, Peter Christie

**Affiliations:** ^1^State Key Laboratory of Coal Resources and Safe Mining, China University of Mining and Technology (Beijing), Beijing, China; ^2^Institute of Ecological and Environmental Restoration in Mining Areas of West China, Xi’an University of Science and Technology, Xi’an, China

**Keywords:** coal mining, grazing, fungal community, *Stipa krylovii*, edaphic variables

## Abstract

Coal mining results in reduced soil quality and makes environments less stable. Soil fungi are suitable indicators of soil quality for monitoring purposes. Here, the objective was therefore to investigate the effects of grazing and mining on the composition of the soil fungal community at the periphery of an opencast coal-mine dump in the Shengli mining area, Xilingol League, Inner Mongolia. A total of 2,110 fungal operational taxonomic units were identified and subdivided into 81 orders and nine categories, based on trophic modes. The sensitive factor to mining was soil pH, and that to grazing were soil nitrate-nitrogen and alkaline phosphatase activity. According to the Pearson correlation and Mantel test, we propose interactions between grazing and coal-mining exist a co-effect and could regulate edaphic variables to alter the behavior of soil fungal community. Moreover, compared with coal-mining, grazing has a greater impact on it. The results provide a basis to further clarify soil fungal ecological functions, and may also contribute to the practice of soil remediation and environmental management in coal-mining areas.

## Introduction

Opencast coal mining still has an important role in both energy production and environmental pollution (Liu and Diamond, 2005), causing soil pollution and degradation (Wang et al., 2014; Du et al., 2018), to threaten soil fertility, vegetation growth, and food safety (Liu et al., 2005; Li et al., 2013). Hussain et al. (2019) report that coal mining contributes trace elements to the soil and ultimately leads to the accumulation of mineral contaminants in food crops and affects human health. The detrimental effects of coal mining can be multiple, involving soil degradation, geological and environmental effects, and loss of biodiversity (Wong, 2003).

Soil fungi are a key component of below-ground biological communities and play an important role in decomposition and nutrient recycling through a number of microbiological and ecological processes (Setala and McLean, 2004; Berg and McClaugherty, 2008; Smith and Read, 2010; Voříšková and Baldrian, 2013; Gaggini et al., 2018). Soil fungi have a widespread ecological distribution and the diversity of the fungal community can be affected by many variables such as precipitation, temperature, root exudates, available nutrients, plant community composition, and cropping pattern (Newsham et al., 2016; Nagati et al., 2018; Zhang et al., 2018; Schmidt et al., 2019; Tervonen et al., 2019).

Previous studies on the effects of coal mining on soil ecosystems have mostly considered soil microorganisms (Baldrian et al., 2008; Wang et al., 2019), enzyme activities (Dimitriu et al., 2010), and soil nutrients (Harris et al., 1993; Mummey et al., 2002). These studies have focused mostly on *in situ* reclamation of coal mining areas. However, there is a lack of such studies in other types of ecosystems such as grasslands.

The grassland environment around a mining area is also affected by anthropogenic interference such as livestock grazing. Grazing affects plant performance directly through the consumption of the above-ground parts of plants, and indirectly via effects on soil microbial community composition, by altering soil properties (Collins et al., 1998; Bardgett and Wardle, 2010). Bardgett and Wardle (2010) reported that grazing can lead to changes in the composition of soil fungal communities. Litter decomposition might also be affected by grazing as a consequence of changes in soil enzyme activities and fungal community composition (Santalahti et al., 2018). Thus, grazing might be used to manipulate vegetation dynamics and the soil microenvironment.

*Stipa krylovii* is a perennial bunchgrass and is representative of typical zonal habitats. *S. krylovii* is native to the Inner Mongolian steppe and is considered to be a constructive species for the ecological restoration of typical degraded grassland. The presence of soil fungi in the microbial community of the steppe soil means that *S. krylovii* plays a key role in sustaining the stability of the grassland ecosystem and its productivity (Collier et al., 2003; Joseph et al., 2008). However, the impact of coal mining and grazing on soil fungal diversity in the rhizosphere of *S. krylovii* and the relationships between the soil fungal communities and edaphic properties remain mostly unexplored.

Here, we tested the hypothesis that soil fungal community composition and function change within a periphery of the opencast coal-mine dump and grazing. In addition, we investigated interactions between edaphic properties and soil fungal community changes. The objectives of this study were: (1) to investigate the diversity and distribution of soil fungal communities in the rhizosphere of *S. krylovii* across different grazing and mining areas, using the high-throughput method; and (2) to analyze how coal mining and grazing exert their effects and to what extent.

## Materials and Methods

### Site Description

The soil studied in the present investigation was located at the periphery of the opencast coal-mine dump in the Shengli mining area, Xilingol League, Inner Mongolia (43°02′–44°52′ N, 115°18′–117°06′ E, ∼988.5 m above sea level). This area is typical semi-arid grassland an average annual temperature of 0–3°C and average annual precipitation of 294.9 mm, with considerable seasonal and diurnal temperature variation. The soil is a meadow chestnut soil (Calciustolls; Soil Survey Staff, 2010). The region is characterized by a typical steppe vegetation and comprises different types of plant community, dominated mostly by the grasses *S. krylovii*, *Aneurolepidium chinense*, and *Achnatherum splendens*.

### Experimental Design and Description of Treatments

Sampling was carried out over an area ∼2 km in different directions from the dump, following a linear design. The mining site was used for strip mining. A special artificial landform dump was formed by the accumulation of surface soil stripping with seven steps and a height of 105 m. The average annual coal output of the mining area is 13.2 × 10^6^ tons, covering an average length of 6.84 km from east to west, an average width of 5.43 km from north to south, and an area of 37.1 km^1^. Three sample plots in the periphery of the opencast coal-mine dump were chosen in each of the three directions (A, B, E): routes A (AR) and B (BR) are grazing areas (main herbivores are sheep and horses), whereas route E (ER) is a restricted grazing area. The distances between the dump and the three sample plots on each route were 100, 900, and 1,900 m (Supplementary Figure 1).

We sampled four replicates areas (each 50 × 50 m, the distance between any two sites exceeded the spatial dependence of most soil variables, <13.5 m) in each of the sample plots with similar slopes, gradients, and altitudes (Marriott et al., 1997). After removing the litter layer, five replicate soil samples were randomly selected from the rhizosphere of native *S. krylovii* individuals selected with an “S” shape and then homogenized combined to provide one composite soil sample per replicate. Rhizosphere soil was collected by separating the soil adhering to the roots using sterilized forceps at a depth of 30 cm at each plot. A total of 36 soil samples (3 routes × 3 plots × 4 replicates) were collected from the study area. Fresh sub-samples (10 g) of each were transferred from the field to the laboratory on ice and then stored at −80°C prior to DNA extraction and high-throughput sequencing. The remaining portion of each sample was sieved (<2 mm), air-dried and stored at 4°C prior to analysis of soil physicochemical properties.

### Soil Sampling

Soil pH and electrical conductivity (EC) were determined using a glass electrode in a soil: water suspension [1:2.5 (w/w)] with a pH meter (PHS-3C; Shanghai Lida Instrument Factory, Shanghai, China) and in a [1:5 (w/w)] suspension with a conductivity meter (DDS-307W; Shanghai Lida Instrument Factory, Shanghai, China), respectively. Soil organic matter (SOM) content was obtained from the percentage organic carbon determined by the dichromate oxidation method in the presence of H_2_SO_4_ (Rowell, 1994). Ammonium nitrogen (NH_4_^+^–N) and nitrate nitrogen (NO_3_^–^–N) were extracted from fresh samples with 1 mol L^–1^ KCl and determined using an auto analyzer (SEAL AutoAnalyzer 3; SEAL Analytical Ltd., Segensworth, United Kingdom; Xiao et al., 2018). Soil available phosphorus (AP) and available potassium (AK) were extracted from fresh samples with 0.5 mol L^–1^ NaHCO_3_ and determined by inductively coupled–plasma emission spectroscopy (ICP-OES, Optima 5300DV, Perkin Elmer, Waltham, MA, United States; Bai et al., 2018). Soil alkaline phosphatase (ALP), acid phosphatase (ACP) and urease (U) activities were determined by the methods described by Tarafdar and Marschner (1994) and Hoffmann and Teicher (1961).

Soil microbial DNA was extracted from 0.1 g of homogenized soil from each fresh sub-sample using a MoBio PowerSoil DNA Isolation Kit (Mo Bio Laboratories, Carlsbad, CA, United States) according to manufacturer’s instructions. PCR amplification was conducted using the internal transcribed spacer (ITS) between the large and small subunit rDNA genes, and specific primers ITS1F (5′-CTTGGTCATTTAGAGGAAGTAA-3′) with barcode and ITS2R (5′-GCTGCGTTCTTCATCGATGC-3′′; Adams et al., 2013). Subsequent PCR amplification thermal cycling consisted of the following steps: initial denaturation at 95°C for 3 min followed by 35 cycles of denaturation at 95°C for 30 s, annealing at 55°C for 30 s, and elongation at 72°C for 45 s, with final elongation at 72°C for 10 min. The PCR products were purified using an AxyPrepDNA gel extraction kit (Axygen, Corning, NY, United States) and mixed in equal density ratios. This was followed by library construction and its assessment with a Qubit 2.0 Fluorometer (Thermo Fisher Scientific, Waltham, MA, United States) and Agilent Bioanalyzer 2100 system (Agilent Corporation, Santa Clara, CA, United States). Purified amplicons were sequenced using an Illumina MiSeq PE300 platform (Illumina Corporation, San Diego, CA, United States). The resulting raw reads were deposited in the NCBI BioProject database (accession number PRJNA682369).

### Analysis

The raw sequences were calculated by QIIME (v.1.7.0^2^, Supplementary Table 1). Sequence analysis was conducted by UPARSE (v.7.0.1001)^2^ and sequences were grouped into operational taxonomic units (OTUs; ≥97% similarity). The representative sequence for each OTU was screened for further annotation to obtain taxonomic information using Unite Database.^3^ The detailed analysis processes for bioinformatics were shown by Bi et al. (2019).

### Statistics

Number of observed species (Sobs), abundance-based coverage estimate (ACE), Shannon–Wiener index and phylogenetic diversity (PD) were calculated and displayed using R software (v.3.5.2^4^) via the “phyloseq” package (McMurdie et al., 2013). OTUs were visualized using histograms or Venn diagrams by R software (v.3.5.2) via the “ggplot2” package. Fungal OTUs were further categorized by annotating the OTUs against the FUNGuild database based on their trophic mode (Nguyen et al., 2016). Those OTUs that could not be assigned to any groups were classified as “unassigned.”

Permutational multivariate analysis of variance (PERMANOVA) and non-metric multidimensional scaling (NMDS) were carried out using R software (v.3.5.2) via the “vegan” package (Oksanen et al., 2007), using the adonis and metaMDS functions, respectively. Quantification of the effect of edaphic variables on soil fungal community composition was calculated using “envfit” from the “vegan” package. Pairwise comparisons of edaphic variables and fungal diversity indices were calculated by use of Pearson correlations. Distances, routes, and fungal community composition were related to each edaphic variable and fungal diversity index by Mantel test. Pearson correlations and Mantel test were visualized using R software via the “ggcor” package. Linear discriminant analysis (LDA) coupled with effect size measurement (LEfSe) analysis was conducted to search for statistically different biomarkers between groups (Segata et al., 2011).

Edaphic variables and fungal diversity indices were assessed by two-way analysis of variance (ANOVA). Comparisons between pairs of means were made using the Tukey’s honestly significant difference tests (*P* < 0.05) using SPSS (v.19.0, IBM, Armonk, NY, United States). Kaleida Graph 4.0^5^ was used for graphical plotting and presentation.

## Results

### Identification of Soil Fungi

A total of 2,110 OTUs and 81 fungal orders were identified, with the predominant orders being Pleosporales (20.77%), Hypocreales (16.15%), unclassified_p__Ascomycota (12.56%), Sordariales (10.78%), Agaricales (10.84%), unclassified_k__Fungi (6.59%), and Eurotiales (3.55%). With reference to the FUNGuild database, OTUs were classified into nine categories, according to trophic modes, with the predominant modes being Saprotroph (28.55%), Pathotroph-Saprotroph-Symbiotroph (21.31%), Pathotroph (9.02%), Pathotroph-Saprotroph (5.93%), Saprotroph-Symbiotroph (1.97%), Symbiotroph (1.47%), Pathotroph-Symbiotroph (0.34%), and Pathogen-Saprotroph-Symbiotroph (0.13%), in addition to Unassigned (31.30%; Supplementary Figure 2). The relative abundance of Pathotroph-Saprotroph was maximum at restricted grazing area (routes E, ER), whereas the relative abundance of Pathotroph-Saprotroph-Symbiotroph was maximum at grazing area (routes A, AR; routes B, BR). The relative abundance of Pathotroph and Symbiotroph generally increased with distances at ER and AR, respectively.

**FIGURE 1:**
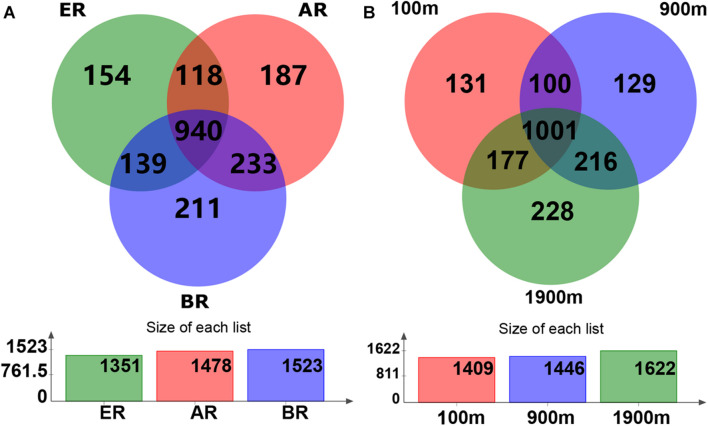
Operational taxonomic unit (OTU) Venn diagram of fungal communities in different routes and at different distances. AR, route **(A)**; BR, route **(B)**; ER, route (E).

Based on the Venn diagram, the common and unique OTU numbers in multiple samples were calculated (Figure 1), with the OTUs, composition similarity and overlap also being intuitively displayed. OTU numbers 1,478, 1,523, and 1,351 on routes A, B, and E were observed, respectively. Of these, 940 (47.4% of the total) OTUs were common to all three routes, while the numbers of unique OTUs on routes A, B, and E were 187 (9.4%), 211 (10.6%), and 154 (7.8%), respectively. Similarly, 1,409, 1,446, and 1,622 OTUs were detected at distances of 100, 900, and 1,900 m from the dump, respectively. Of these, 1,001 (50.5%) OTUs were common to all three distances while the numbers of unique OTUs at 100, 900, and 1,900 m were 131 (6.6%), 129 (6.5%), and 228 (11.5%), respectively.

### Soil Properties Analysis

Soil organic matter also differed significantly, depending on the sampling sites (*P* < 0.05, Table 1), with a maximum of 37.1 mg g^–1^ at 1,900 m of routes E (ER) and with a minimum of 23.2 mg g^–1^ at 100 m of routes A (AR). Soil NH_4_^+^–N concentrations at 900 and 1,900 m of AR were significantly higher than concentrations at other sampling sites, while the lowest concentration was recorded at 1,900 m of routes B (BR, *P* < 0.05). Soil NO_3_^–^–N concentration at 900 m of AR was also significantly higher than at other sampling sites (*P* < 0.05). AP concentrations at 900 and 1,900 m of ER were significantly higher than those at the other sites, and the minimum concentration was 7.17 mg kg^–1^ at 100 m of BR and the maximum reached 10.4 mg kg^–1^ at 1,900 m of ER. Soil available potassium (AK) concentrations ranged from 219 to 292 mg kg^–1^ at different sampling sites, with values at 1,900 m of AR and 1,900 m of ER being significantly higher than those at other sampling sites (*P* < 0.05).

**TABLE 1:** Mean values (±SD) of edaphic variables under different distances and routes.

Variable^†^	AR			BR			ER			Route	Distance	R × D
	100 m	900 m	1900 m	100 m	900 m	1900 m	100 m	900 m	1,900 m			
pH	6.94ab^§^	6.66d	6.79bcd	6.87abc	6.75cd	6.81bcd	7.00a	6.90abc	6.81bcd	ns	***	ns
EC (μs cm^–1^)	68.93c	89.68a	81.05b	62.98d	50.58e	55.85e	68.73c	70.6c	80.93b	***	*	**
SOM (mg g^–1^)	23.16e	27.21de	33.95abc	33.48abc	30.91bcd	33.28abc	34.58ab	29.15cd	37.06a	***	*	**
NH^4+^–N (mg L^–1^)	1.24b	1.79a	1.78a	1.02c	0.69d	0.67d	0.91c	1.18b	0.91c	***	*	***
NO^3–^–N (mg L^–1^)	1.635f	3.78a	1.30g	2.14e	0.78h	0.83h	2.99d	3.19c	3.55b	***	**	**
AP (mg kg^–1^)	8.33b	8.40b	8.57b	7.17c	7.69bc	8.53b	7.62bc	9.60a	10.41a	**	***	***
AK (mg kg^–1^)	261.11b	227.97de	292.35a	256.59b	219.14e	237.94cd	231.8de	251.73bc	282.79a	*	ns	**
ACP (μg g^–1^ h^–1^)	306.3a	250.07b	244.67b	317.97a	296.1a	291.93a	297.2a	251.43b	289.7a	**	**	ns
ALP (mg kg^–1^)	263.67d	237.63e	246.57e	282.9c	267.03d	259.33d	310a	296.9b	297.37b	***	***	ns
U (mg kg^–1^)	60.77b	45.36d	58.07b	44.71d	40.77e	53.07c	53.64c	58.23b	69.3a	***	*	*

*^†^EC, electrical conductivity; SOM, soil organic matter; AP, available phosphorus; AK, available potassium; ACP, acid phosphatase; ALP, alkaline phosphatase; U, urease.*

*^§^ Values within a column followed by the same letter are not significantly different at *P* ≤ 0.05.*

*, ** and *** indicate statistical significance at *p* < 0.05, 0.01, and 0.001, respectively. ns, not significance.

The ACP activities at 900 m of ER, and at 900 and 1,900 m of AR were significantly lower than at other sampling sites, while the greatest activity was recorded at 100 m of BR, with a maximum value of 306 μg g^–1^ h^–1^ (*P* < 0.05). ALP activity ranged from 238 to 310 μg g^–1^ h^–1^, the maximum value being recorded at 100 m of ER which was significantly higher than that at other sampling sites (*P* < 0.05). U activity at 1,900 m of ER was significantly higher than at other sampling sites (*P* < 0.05), with the lowest value occurring at 900 m of BR.

### Fungal Communities

**FIGURE 2:**
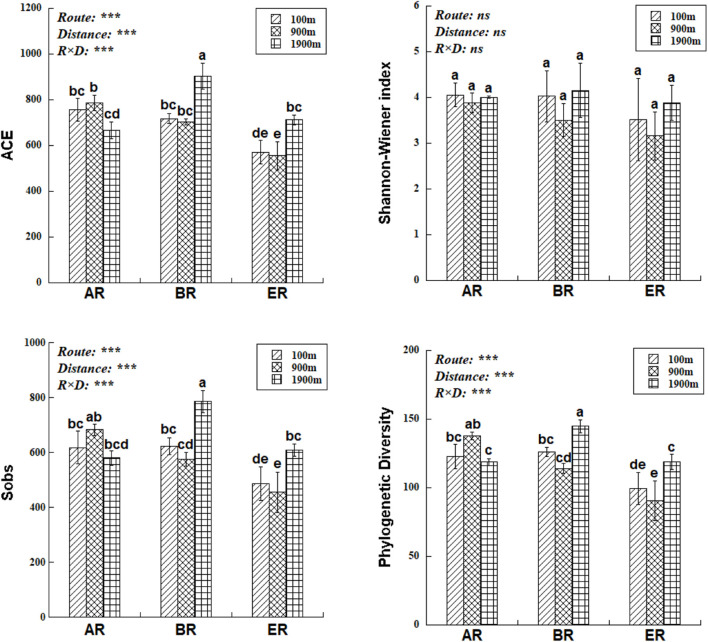
Differences in fungal abundance-based coverage estimate (ACE), number of observed species (Sobs), phylogenetic diversity (PD) and Shannon–Wiener index at different distances and routes. Two-way ANOVA reveals the effect of route (R), distance (D), and their interactions (R × D) on fungal diversity indices (ns, not significant, *P* ≥ 0.05; ***P < 0.001). Error bars represent standard deviation. Bars followed by lowercase letters are significantly different among distances and routes (*P* < 0.05).

**FIGURE 3:**
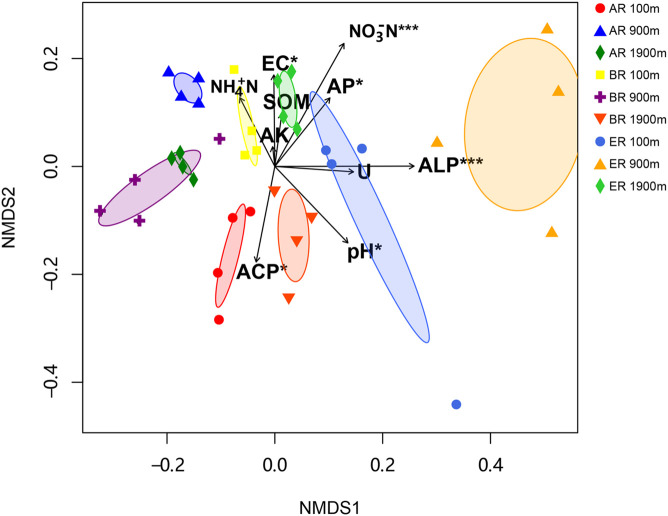
Non-metric multidimensional scaling (NMDS) of fungal community (stress = 0.249). Significant variables NO_3_^–^ –N (NO_3_^–^ –N; *R*^2^ = 0.430, *P* < 0.001), alkaline phosphatase (ALP; *R*^2^ = 0.419, *P* < 0.001), acid phosphatase (ACP; *R*^2^ = 0.204, *P* = 0.026), electrical conductivity (EC, *R*^2^ = 0.180, *P* = 0.034) and pH (*R*^2^ = 0.242, *P* = 0.012) are fitted onto the NMDS graph based on the results of “envfit” function analysis. The arrows represent fitted vectors of edaphic variables with distribution significantly correlated with fungal community composition (*P < 0.05, ***P < 0.001). SOM, soil organic matter content; AK, available potassium; AP, available P; Urease, activity of soil urease.

The range of Sobs of BR, from 622 to 785, was higher than those of AR (580–683) and ER (455–609; Figure 2). In the BR and ER samples the lowest values of ACE, number of observed species (Sobs) and PD occurred at 900 m, and the highest at 1,900 m. The diversity indices of ACE, Sobs, and PD were affected by route, distance and the interaction between these two factors, while the Shannon–Wiener index was unaffected by them. PERMANOVA analysis indicates that soil fungal community composition was significantly affected by different routes (pseudo-*F* = 4.455, *P* = 0.001) and distances (pseudo-*F* = 3.029, *P* = 0.009). NMDS shows that the fungal community composition was significantly affected by different routes and distances (Figure 3). Fungal community composition was significantly affected by NO_3_^–^–N concentration (*P* < 0.001), ALP activity (ALP, *P* < 0.001), ACP activity (ACP, *P* = 0.026), EC (*P* = 0.034) and pH (*P* = 0.012).

### Correlation Analysis

**FIGURE 4:**
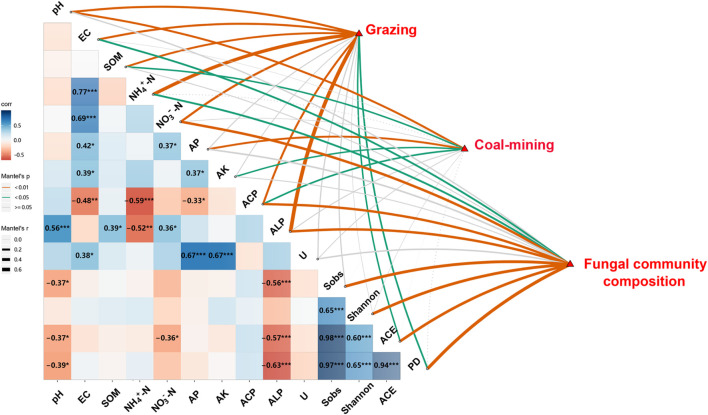
Relationships among distances, routes, edaphic variables, fungal diversity indices, and composition of fungal community. Pairwise comparisons of edaphic variables and fungal diversity indices with a color gradient denoting Pearson’s correlation coefficient (*P < 0.05, **P < 0.01, ***P < 0.001). Distances, routes, and fungal community composition were related to edaphic variables and fungal diversity indices by Mantel test. SOM, soil organic matter content; EC, electrical conductivity; AK, available potassium; AP, available P; Urease, activity of soil urease; ALP, activity of alkaline phosphatase; ACP, activity of acid phosphatase; Shannon, Shannon–Wiener index; PD, phylogenetic diversity.

The Pearson correlation and Mantel test showed significant relationships among route, distance, diversity and composition of fungal communities, and edaphic variables (Figure 4). Distance had significant effects on soil pH, SOM, AP, AK, and ACP (*P* < 0.05). Route had significant effects on edaphic variables, ACE, and PD (*P* < 0.05), whereas edaphic variables had close relationships with the diversity and composition of fungal communities. Soil EC, NH_4_^+^–N, and NO_3_^–^–N concentration and ACP and ALP significantly influenced fungal community composition (*P* < 0.05). Soil pH and ALP were significantly and negatively correlated with Sobs, ACE, and PD. Soil NO_3_^–^–N was significantly and negatively correlated with ACE (*P* < 0.05).

### Microbial Communities With Statistically Significant Differences

**FIGURE 5:**
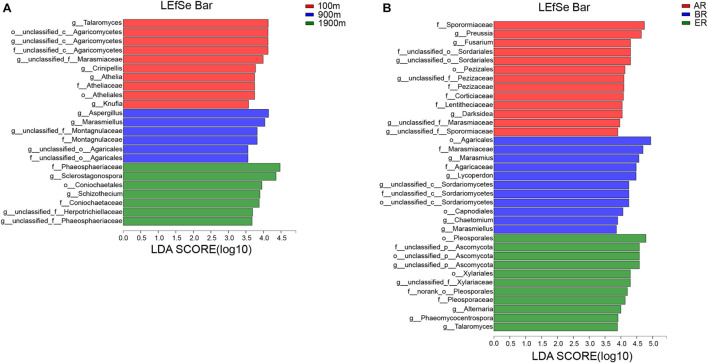
Indicator fungi with LDA scores of 3.5 or greater in fungal communities associated with different distances **(A)** and routes **(B)**.

LEfSe identifies features that are statistically different from order to genus (Kruskal–Wallis sum-rank test, *P* < 0.05). LDA scores of groups were confirmed by LEfSe (Figure 5). At 100 m, four groups were significantly predominated (Figure 5A), namely unclassified_c_Agaricomycetes (from order to genus and *Crinipellis*), Atheliales (from order to genus), *Talaromyces* and *Knufia*. At 900 m, three groups were significantly predominated (Figure 5A), unclassified_c_Agaricales (from family to genus and *Marasmiellus*), Montagnulaceae (from family to genus) and *Aspergillus*. At 1,900 m, five groups were significantly predominated (Figure 5A), namely Coniochaetales (from order to family), Phaeosphaeriaceae (from family to geuns), *Sclerostagonospora*, *Schizothecium*, and unclassified _f_Herpotrichiellaceae (genus). In AR, 11 groups were significantly predominated (Figure 5B), namely Pezizales (from family to genus) unclassified_o_Sordariales (from family to genus), Sporormiaceae (family), Corticiaceae (family), Lentitheciaceae (family), unclassified_f_Marasmiaceae (genus), unclassified_f_Sporormiaceae (genus), *Darksidea*, *Preussia*, and *Fusarium*. In BR, three groups were significantly predominated (Figure 5B), namely unclassified _c_Sordariomycetes (from order to genus, its genus *Chaetomium*), Agaricales (from order to family, its family Marasmiaceae and genus *Marasmiellus, Lycoperdon*, and *Marasmius*), Capnodiales (order). In ER, four groups were significantly predominated (Figure 5B), namely unclassified_p_Ascomycota (from order to genus), Pleosporales (from order to family, and its genus Alternaria and Phaeomycocentrospora), Xylariales (from order to family) *Talaromyces*.

## Discussion

### Impact of Coal Mining on Fungal Communities

The data here suggest that the diversity of the fungal community had its maximum values at the sampling plots farthest from the coal mining dump, but there was no linear relationship between diversity and distance. The relative abundance of Symbiotroph increased with distances at grazing area (AR), correlated with the establishment of partner species in the stages. Symbiotic fungi affect ecosystem functions by influencing plant community assembly and increasing plant productivity (Bever et al., 2010, 2012). A potentially mutually beneficial relationship with plants, can be formed by symbiotic fungi, such as mycorrhizal fungi, enabling the host plants to enter otherwise inaccessible habitats, and increasing their competitiveness (Bever et al., 2010).

The fungal community composition and edaphic variables were significantly affected by the distance from the dump, whereas edaphic variables significantly influenced fungal diversity indices by correlation analysis. The effect of distance from the site of coal mining activity on fungal diversity may have occurred through changes in edaphic variables. This finding is similar to the observations of Du et al. (2018) who reported that the geochemical composition of the soil is greatly changed at sites close to mining areas due to rapid industrialization and coal production.

Soil pH was an important variable controlling fungal community structure (Rousk et al., 2010; Rousk and Bååth, 2011; Taylor et al., 2014). The availability of nutrients to most plant species is maximum between pH values 6.0 and 7.5, and an increase in soil pH value can limit the availability of nutrients (Asghar et al., 2008). Here, soil pH was significantly higher at 100 m than at 900 or 1,900 m, and was negatively correlated with soil fungal diversity indices, indicating that soil fungi are sensitive to changes in soil pH, with high pH possibly inhibiting the diversity of fungal community to a certain extent by limiting the availability of soil nutrients. Soil enzyme activities may be an essential factor influencing both the metabolism of soil nutrients and the composition of the fungal community (Atul-Nayyar et al., 2009). Correlation analysis showed that soil available P was significantly related to distance, and activity of ACP was significantly related to distance and fungal community composition. Thus, the increase in numbers of fungal species with increasing distance from the dump might help to promote soil metabolic activity, thereby effectively increasing soil available P content.

### Impact of Grazing on Fungal Communities

Grazing was previously found to reduce soil microbial respiration and activity, an observation which was suggested to result in decreased decomposition rate (Väre et al., 1996; Stark et al., 2003). Here, the diversity of fungi in grazed areas increased relative to that in areas with restricted grazed, with a community composition pattern dominated by saprotrophs being formed. Moreover, grazing was the main factor affecting the distribution of the fungal community in the research area, with the diversity of soil fungi possibly increasing in response to moderate grazing. Our data are consistent with the conclusions of Whittaker (1960) and Cline et al. (2017), but contradictory to the findings of Santalahti et al. (2018), who concluded that grazing had a significant impact on soil fungal community structure but had no significant effect on soil fungal diversity.

In respect of NMDS, the composition of the fungal community was significantly affected by grazing. Grazing likely modulates the soil fungal community by altering the competitive interactions between dominant fungi and releasing/suppressing subordinate fungi (Eldridge et al., 2017), or by directly affecting soil fungi through changing resource availability by the production of dung and urine (McNaughton et al., 1997; Liu et al., 2015; Yang et al., 2019).

Grazing can also directly impact microbes through altering soil physical and chemical properties (Yang et al., 2019). The composition of soil microbial communities has been reported to be correlated with soil NO_3_^–^–N under different grazing regimes in soil of the Loess Plateau and to be correlated with soil pH in grazed grassland on the Tibetan Plateau (Li et al., 2015; Chen et al., 2017). These findings are similar to those from our study on fungi. Soil NO_3_^–^–N concentration in grazing areas was significantly lower than in restricted grazed areas, most likely due to reduced biomass of aboveground vegetation by herbivory, and then the amount of nitrogen transferred to the soil decreased. Results of the Pearson correlation show that soil NO_3_^–^–N concentration was significantly negative correlated with fungal community diversity, suggesting that grazing might indirectly caused the change in fungal communities.

Fungi are the most efficient decomposers of organic matter and produce a wide range of extracellular enzymes (Santalahti et al., 2018). Enzyme activities have also been reported to change in response to substrate availability in soil microorganisms as a result of grazing (Stark and Väisänen, 2014). Plants can absorb inorganic P by the phosphatase hydrolysis of organic P (He et al., 2011). Here, ALP activity was significantly higher in the restricted grazing area than in the grazed area, and was shown to be a major factor affecting the distribution and composition of the fungal community, demonstrating that grazing might reduce phosphatase activity and hence indirectly weaken the cycling and utilization of soil P, allowing for the regulation of the composition of the fungal community.

## Conclusion

Here, we demonstrate the detailed soil fungal community and soil nutrients at the periphery of an opencast coal-mine area. The diversity of the fungal community had its maximum values at the sampling plots farthest from the coal mining dump, but without linear relationship between diversity and distance. The sensitive factor to mining was soil pH. The sensitive factors to grazing were soil nitrate-nitrogen (NO_3_^–^–N) and ALP activity. Interactions between grazing and coal mining show a coupling effect in the behavior of soil fungal communities. Future studies investigating the ecological functions of soil fungi would increase our understanding of the role of soil fungal communities in grassland coal-mining areas.

## Data Availability Statement

The original contributions presented in the study are publicly available. This data can be found here: https://www.ncbi.nlm.nih.gov/bioproject/ (accession number PRJNA682369).

## Author Contributions

YB and LX conceived and designed the experiments. LX and XL performed the experiments. LX and KW analyzed the data and language editing with the help of PC. All authors contributed to the article and approved the submitted version.

## Conflict of Interest

The authors declare that the research was conducted in the absence of any commercial or financial relationships that could be construed as a potential conflict of interest.

## Publisher’s Note

All claims expressed in this article are solely those of the authors and do not necessarily represent those of their affiliated organizations, or those of the publisher, the editors and the reviewers. Any product that may be evaluated in this article, or claim that may be made by its manufacturer, is not guaranteed or endorsed by the publisher.

## Footnotes

1
http://www.drive5.com/uparse/

2
http://qiime.org/

3
https://unite.ut.ee/

4
https://www.r-project.org/

5
http://www.synergy.com/wordpress_650164087/
