# Supplementary material for: Soil Fungal Community in Grazed Inner Mongolian Grassland Adjacent to Coal-Mining Activity
Source: Front Microbiol. 2021 Sep 17;12:718727. doi: 10.3389/fmicb.2021.718727 (PMC8484957; doi:10.3389/fmicb.2021.718727)
Supplement: Supplementary file 1 [file Data_Sheet_1.docx]

Supplementary Material

# Supplementary Figures and Tables

## Supplementary Figures


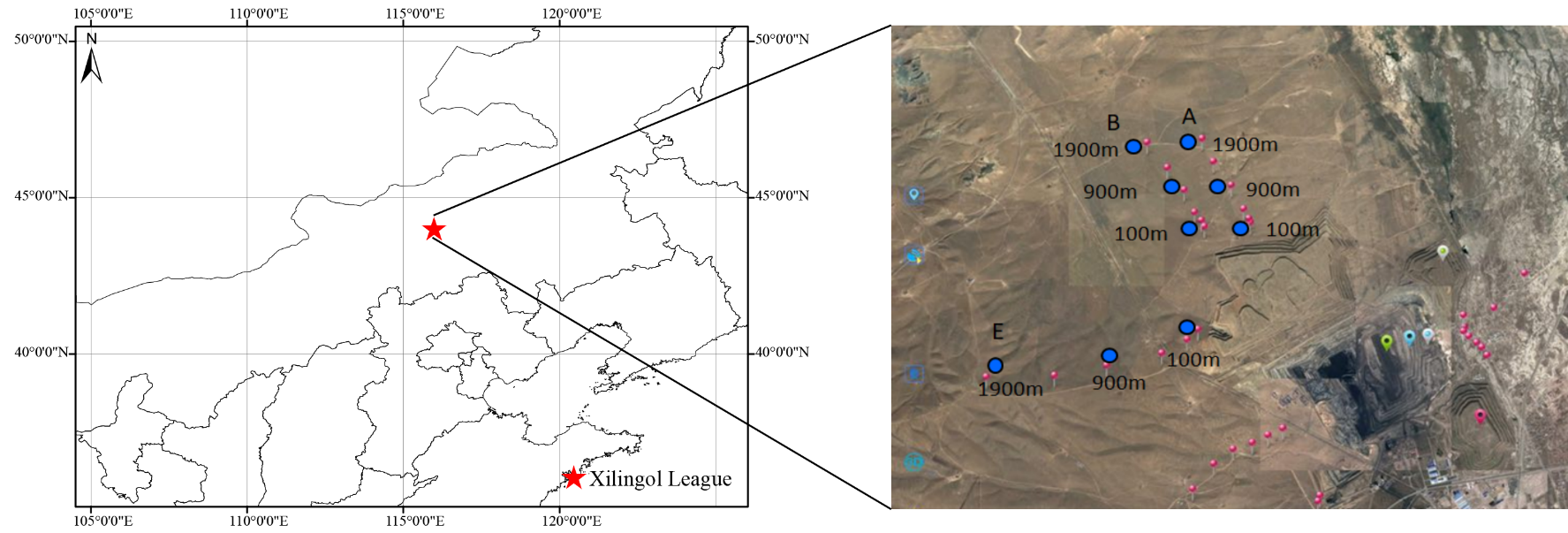
**Supplementary Figure 1.** Map showing the location of the study area


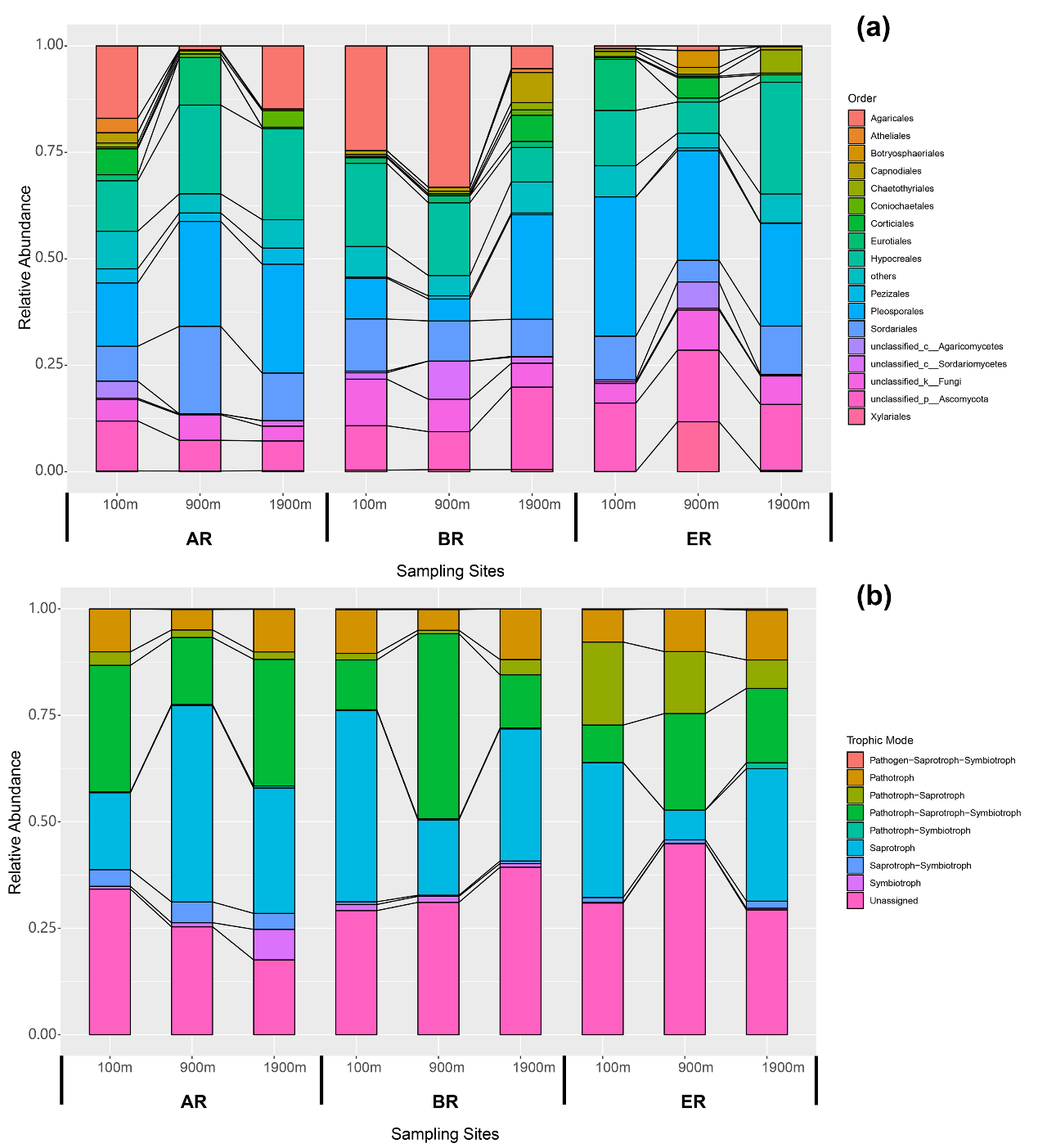
**Supplementary Figure 2.** Column chart of the proportion of soil fungal order. AR (route A), BR (route B), ER (route E).

## Supplementary Tables

**Supplementary Table 1.** The information of sequencing results under different distances and routes.

| Variable | AR | | |  | BR | | |  | ER | | |
| --- | --- | --- | --- | --- | --- | --- | --- | --- | --- | --- | --- |
|  | 100m | 900m | 1900m |  | 100m | 900m | 1900m |  | 100m | 900m | 1900m |
| total read number | 50612.25b^†^ | 70759.5a | 57338ab |  | 60395ab | 57530.5ab | 70882.5a |  | 61991.25ab | 60789.5ab | 63180.25ab |
| Bases  (bp) | 14057425.5a | 18179752.75a | 15370021.5a |  | 17313677.75a | 16131653.25a | 19464869.25a |  | 16091867a | 16633078.5a | 16178856a |
| Average Length | 277.83ab | 257.03c | 268.09abc |  | 285.63a | 279.5a | 274.62abc |  | 259.9bc | 273.62abc | 256.15c |

^†^Values within a column followed by the same letter are not significantly different at *P*≤0.05.
